# Whole-Genome Analysis of a Historical Strain of *Xanthomonas citri* pv. *citri* Reveals Structural Variations

**DOI:** 10.3390/ijms27020702

**Published:** 2026-01-09

**Authors:** Wenting Li, Li He, Bin Guan, Xiaoxue Zeng, Zheng Zheng, Jian He

**Affiliations:** 1 Institute of Horticulture, Sichuan Academy of Agricultural Sciences, Chengdu 610066, China; wentingl2015@163.com (W.L.); heli@scsaas.cn (L.H.); gullett@163.com (B.G.); zengxiaoxue@hotmail.com (X.Z.); 2 National-Local Joint Engineering Laboratory of Citrus Breeding, Chengdu 610066, China; 3 Key Laboratory of Horticultural Crops Biology and Germplasm Enhancement in Southwest China, Ministry of Agriculture and Rural Affairs, Chengdu 610066, China; 4 College of Plant Protection, South China Agricultural University, Guangzhou 510642, China

**Keywords:** historical specimens, *Xanthomonas citri* pv. *citri*, genome analysis, structural variations

## Abstract

Plant disease specimens are invaluable resources for investigating the origin and spread mechanisms of plant pathogenic microorganisms. Citrus canker, caused by *Xanthomonas citri* pv. *citri* (*Xcc*), is one of the most devastating bacterial diseases in citrus production. Here, we report the complete genome sequence of *Xcc* strain GD82, isolated from Guangdong Province during the early outbreak stage in the 1980s. Comparative analysis with modern genomes revealed key differences in structural variations, functional single-nucleotide polymorphisms (SNPs), and phage-related fragments, suggesting potential associations between insertions/deletions (InDels) and pathogenicity or environmental adaptation. This study provides critical insights into the evolutionary trajectory of *Xcc* and the epidemiological dynamics of citrus canker in China.

## 1. Introduction

Citrus canker, caused by *Xanthomonas citri* pv. *citri* (*Xcc*), is a major bacterial disease that inflicts severe economic losses on the global citrus industry, continuously threatening production security and trade stability [1]. Clarifying its origin, spread, and evolutionary patterns is fundamental for developing effective control strategies.

Plant disease specimens serve as invaluable historical archives, playing an irreplaceable role in tracing the geographical origin and dissemination routes of pathogens. Studies based on early specimens once suggested that the disease might have originated in Southeast Asia or India [2,3]. With advances in molecular technology, analyzing nucleic acids preserved in specimens has become feasible. For instance, research on historical specimens of potato late blight confirmed its causal agent [4], and genetic analysis of historical *Xcc* strains revealed the introduction pathway of the Florida, USA outbreak [5,6]. However, DNA in historical specimens is often severely degraded, and early studies were largely limited to PCR-based detection of specific genes [7,8,9], constraining the ability to obtain information at the whole-genome level.

In recent years, innovations in high-throughput sequencing technologies, particularly the application of long-read sequencing, have greatly advanced pathogen genomics. Whole-genome sequencing not only enables efficient pathogen identification and typing [10,11] but also allows the acquisition of high-quality genomic data from historical specimens, thereby enabling the analysis of pathogen evolutionary dynamics across time. This approach has been successfully applied to reconstruct the dissemination history and adaptive evolution of various crop pathogens, including tracing global spread events of *Xcc* [12,13], and revealing historical shifts in the host range of other diseases [14,15]. These studies highlight the powerful capability of comparing historical and modern genomes in uncovering key evolutionary events such as gene gain, loss, and structural variation [16].

Nevertheless, despite the global expansion of *Xcc* genomic resources, data from Chinese strains—particularly historical outbreak strains—remain markedly underrepresented in public databases [13,17,18]. This gap is especially critical given China’s central role in both global citrus cultivation and the historical geography of the disease. As one of the world’s largest citrus producers, China contributes substantially to global output, and epidemiological and historical evidence suggests that East or Southeast Asia, a region encompassing China, may represent a source area or a historical dissemination hub for citrus canker [2,19]. Existing analyses indicate that the poor representation of Chinese *Xcc* strains in public databases may lead to an underestimation of their genetic diversity and limits in-depth understanding of the disease’s evolutionary history in this region. Specifically, the lack of genomic data from historical strains collected during key early outbreak periods in China hinders fine-scale mapping of *Xcc*’s local adaptation and evolutionary trajectory domestically, while also constraining accurate assessment of the status of Chinese strains within the global evolutionary framework. This scarcity not only restricts a comprehensive understanding of the population diversity, evolutionary pathways, and local adaptation of *Xcc* in a key citrus-producing region but also represents a significant blind spot in reconstructing the pathogen’s complete global spread and evolutionary history. Therefore, generating and analyzing high-quality genomes from historical Chinese isolates, such as the GD82 strain obtained from the 1980s Guangdong outbreak, is crucial for filling this pivotal knowledge gap and constructing a more complete picture of the pathogen’s global evolution.

To address this critical gap, this study selected the historical strain GD82, isolated from Guangdong Province during the early epidemic stage of citrus canker in the 1980s. By optimizing methods for extracting degraded DNA from formalin-fixed specimens and utilizing modern sequencing platforms, we successfully completed the whole-genome sequencing and assembly of this strain. Through systematic analysis of the GD82 genome and comparison with strains from different periods and regions worldwide, this study aims to (1) enrich the genomic database of Chinese *Xcc*, particularly for historical strains; (2) reveal the unique genomic features of this historical strain (such as structural variations, prophage elements, etc.); and (3) investigate the evolutionary position and potential adaptive mechanisms of early epidemic strains in China from a genomic perspective, thereby providing key genomic evidence for elucidating the evolutionary trajectory and epidemic patterns of citrus canker in China.

## 2. Results

### 2.1. Characterization of Gd82 Strain DNA from Historical Specimen

**Figure 1:**
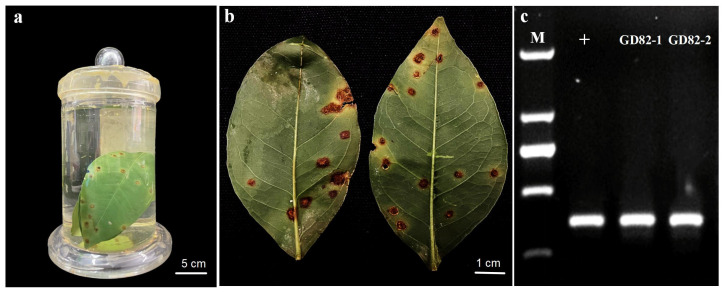
Canker symptom and PCR detection result of a historical formalin-fixed Citrus sinensis specimen. (**a**). Formalin-fixed citrus samples collected in Guangzhou city in 1982. (**b**). Formalin-fixed citrus leaves showed canker erumpent lesions. (**c**). Agarose gel electrophoresis result showed expected PCR product sizes for *Xcc* in the GD82 DNA samples. Note: M, DNA ladder (top to bottom in bp: 2000 bp, 1000 bp, 750 bp, 500 bp and 250 bp). “+”: positive control, GD82-1 and GD82-2, two DNA samples extracted from GD82 sample.

The historical specimen exhibited reddish-brown lesions surrounded by a yellow-green halo. After extracting genomic DNA from the *Xcc*-infected immersion specimen, the concentration of the GD82 strain DNA sample was measured as 25 ng/μL with an A260/A280 ratio of 1.85. Conventional PCR amplification yielded a target band of 413 bp (Figure 1). Real-time PCR using a fluorescent dye detected the GD82 strain DNA sample with a Ct value of 17.39.

### 2.2. Whole-Genome Sequencing Results of Gd82 Strain

Sequencing of the GD82 sample generated 102,741,744 reads with an average length of 150 bp. After removing plant genomic sequences, 102,592,806 reads remained, which were assembled into a genome sequence of 5,136,479 bp. When aligned to the reference genome of strain 306 (38,352,851 reads), the GD82 genome showed mapping to the reference plasmids: 917,547 bp aligned to pXAC33, 1,513,635 reads to pXAC64, 805,169 reads to pXAC47, and 1,754,451 reads to pLJ207-7.1 (Table 1).

### 2.3. Basic Characteristics of the Gd82 Strain Whole Genome

**Figure 2:**
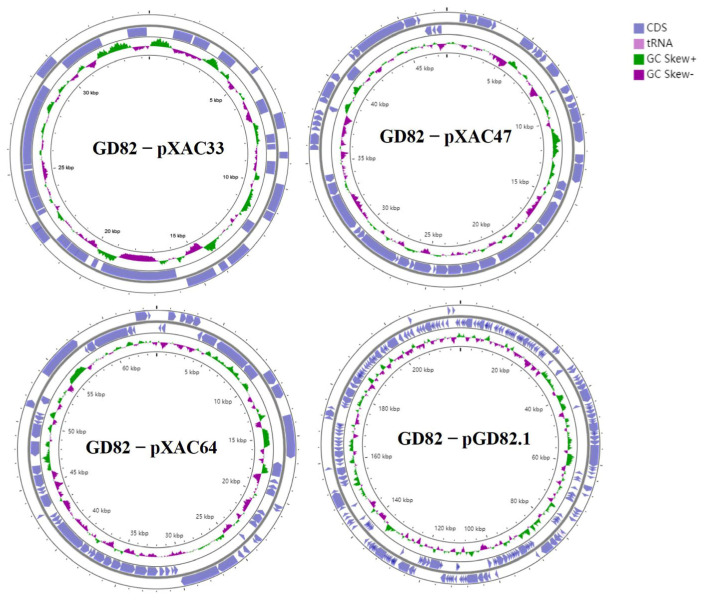
Schematic diagram of 4 circular plasmids of GD82 from historical specimens.

The genome of the *Xcc* historical strain GD82 has a length of 5,197,184 bp with a GC content of 64.8%, and contains four circular plasmid sequences (32,377 bp, 63,972 bp, 47,689 bp, and 219,560 bp) (Figure 2). Annotation results revealed that the GD82 genome contains 4763 coding sequences (CDS) and 100 RNA genes, including 54 tRNA and 6 rRNA genes (5S, 16S, 23S) (Table 2).

### 2.4. Whole-Genome Comparative Analysis

**Figure 3:**
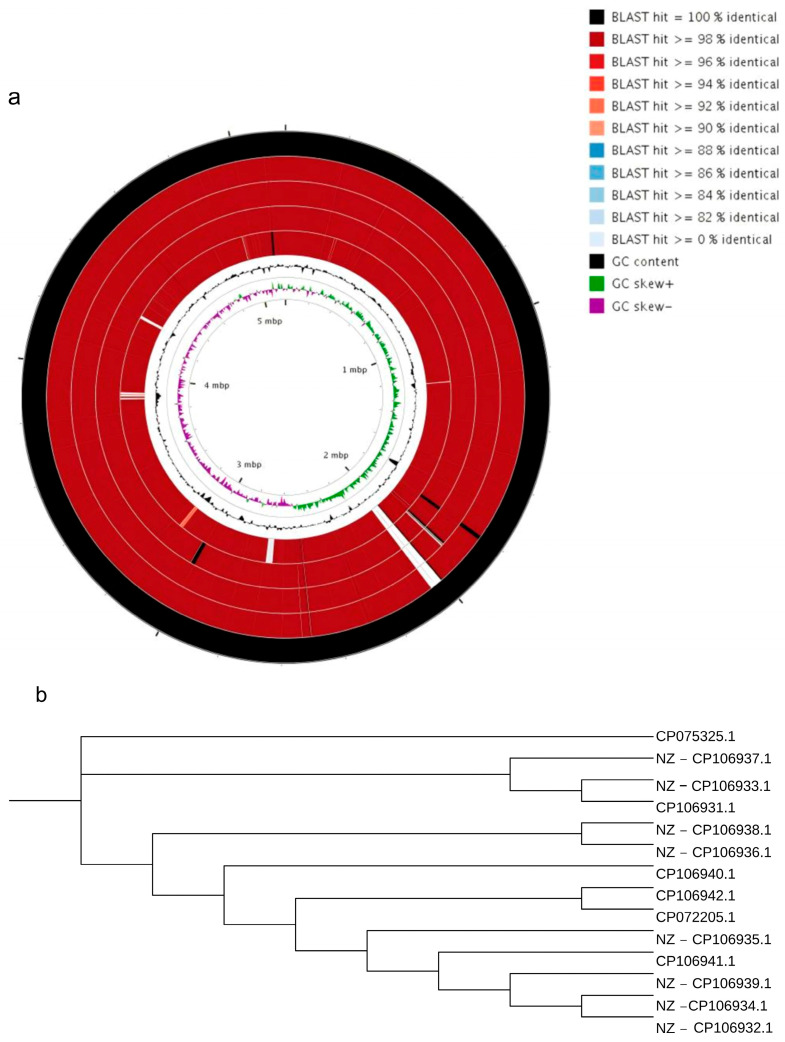
Whole-genome (**a**) and phylogenetic tree analysis (**b**). Note: from outer ring to inner ring, the strains were 306, WG16, GD82, MN11, U17, Aw12879.

Aw12879, MN11, U17, WG16, and 306 were used as reference genomes. The alignment results between the GD82 strain and other strains are shown in Figure 3a. The alignment results indicate that the Aw12879 strain exhibits significant differences from other strains. Specifically, GD82 has the highest similarity (99.99%) with WG16 and the lowest similarity (96.47%) with Aw12879. Additionally, there are deletions and insertions in the sequences.

To understand the genetic differences between our strain and the 13 previously reported historical genomes (Supplementary Table S1), we constructed a phylogenetic tree. The resulting tree revealed that CP075325.1 is distinguished from all other sequences, indicating that CP075325.1 diverged evolutionarily from the other genomes at an earlier stage (Figure 3b).

### 2.5. Analysis of Insertion/Deletion (Indel) Fragments in the Genome of Strain Gd82

**Figure 4:**
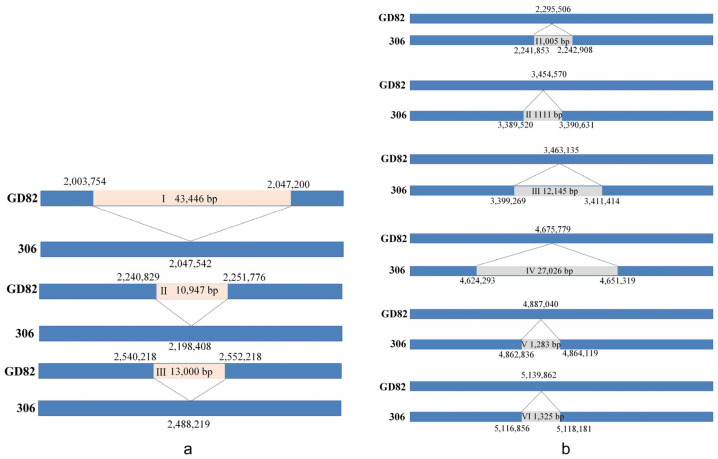
The whole genome sequence of strain GD82 was compared with that of strain 306. Note: (**a**) Three insertions on strain GD82. The orange boxes (I, II, III) represent the inserted fragment; (**b**) Six missing fragments on strain GD82. The gray boxes (I, II, III, IV, V, VI) represent missing fragments.

By comparing the genome of the GD82 strain with that of the *Xcc*306 strain, it was found that there were three large insertions of 43,446 bp, 10,947 bp, and 13,000 bp in the chromosomal region of the GD82 strain genome (Figure 4a). The corresponding insertion site for the 43,446 bp insert sequence on position I of the GD82 strain genome was 2,047,542 to 2,047,543 on the *Xcc*306 genome, which carried 39 hypothetical proteins, 8 phage-related proteins, 5 DUF family proteins, 3 helix-turn-helix domain proteins, and other family genes (Supplementary Table S1). The corresponding insertion site for the 10,947 bp insert sequence on position II was 2,198,408 to 2,198,409 on the *Xcc*306 genome, which contained two methyl chemokines KJA71_RS10015 and KJA71_RS10040, one hypothetical protein KJA71_RS10020, one flagellin KJA71_RS10030, and one fusion protein KJA71_RS10035. The insertion site corresponding to the 13,000 bp insert sequence on position III is located at 2,488,219 to 2,488,220 on the *Xcc*306. This sequence carries six hypothetical proteins KJA71_RS11235, KJA71_RS11240, KJA71_RS11275, KJA71_RS11285, KJA71_RS11290 KJA71_RS11295, five DUF family proteins KJA71_RS11255, KJA71_RS11265, KJA71_RS11260, KJA71_RS11245, KJA71_RS11280, one replication protein KJA71_RS11225, one DNA ligase KJA71_RS11230, one toxin family protein KJA71_RS11250 and one transporter protein KJA71_RS11270 (Supplementary Table S2).

The genome of the GD82 strain has six fragments of deletion of 1005 bp, 1111 bp, 12,145 bp, 27,026 bp, and 1283 bp in the chromosomal region (Figure 4b). Positions I, II, V, and VI have a deletion of 1005 bp, 1111 bp, 1283 bp, and 1325 bp, respectively. The annotation results indicate that positions I, II, V, and VI carry a transposon-related gene *XAC_RS09765*, *XAC_RS14670*, *XAC_RS20845*, and *XAC_RS21830*. The annotation results of the position III sequence show a hypothetical protein XAC_RS01495. The annotation results of the position IV sequence show an integrase gene *XAC_RS19845* and a helix-turn-helix gene *XAC_RS19930*.

### 2.6. Analysis of Gd82 Strain-Associated Plasmids

**Figure 5:**
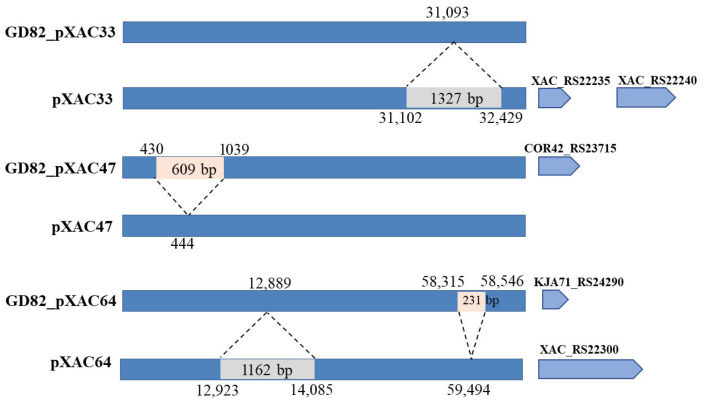
Comparison of plasmid sequences of the GD82 strain with other plasmids. Note: The orange box represents the inserted fragment and the gray box represents the missing fragment.

Compared to plasmid pXAC33, the GD82_pXAC33 plasmid exhibited a 1327 bp deletion (corresponding to positions 33,102–32,429 in pXAC33). Annotation revealed that this deleted region encoded one hypothetical protein and one AAA family ATPase gene (*XAC_RS22235* and *XAC_RS22240*). Compared to plasmid pXAC47, GD82_pXAC47 contained a 609 bp insertion (at position 444–445 in pXAC47), which was annotated as encoding one hypothetical protein. Compared to plasmid pXAC64, GD82_pXAC64 had a 1162 bp deletion (spanning positions 12,923–14,085 in the reference sequence), annotated as a UPF0149 family protein. Additionally, GD82_pXAC64 harbored a 231 bp insertion (at positions 58,315–58,546), partially encoding a TAL effector repeat-containing protein (Figure 5).

### 2.7. Genome Annotation of Gd82 Strain

(1) Analysis and validation of prophages in the immersion specimen of the GD82 strain

**Figure 6:**
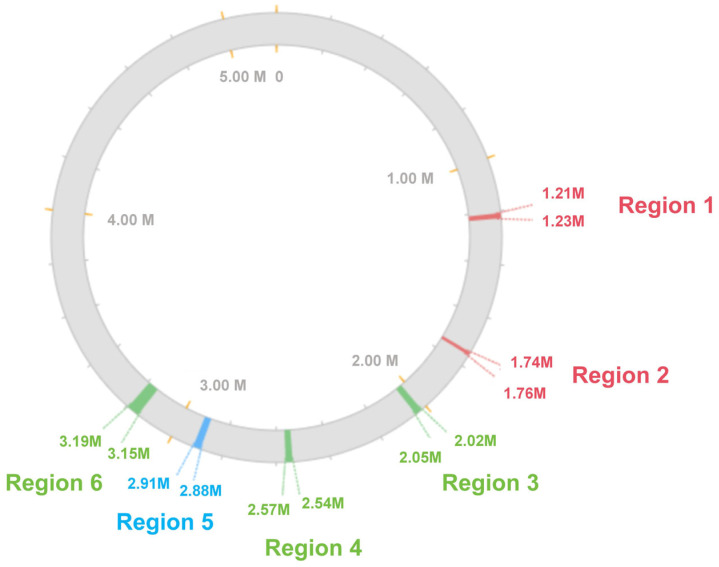
Distribution of the prophage region in the genome of strain GD82. Note: Green region represents intact prophage, blue region questionable prophage, red region represents incomplete prophage.

**Figure 7:**
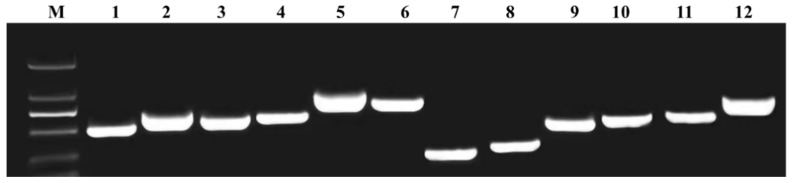
Electrophoresis results of PCR amplification products in the prophage region of strain GD82. Note: M: 2000 bp DNA ladder 1: Left side of prophage region 1, 2: Right side of prophage region 1, 3: Left side of prophage region 2, 4: Right side of prophage region 2, 5: Left side of prophage region 3, 6: Right side of prophage region 3, 7: Left side of prophage region4, 8: Right side of prophage region 4, 9: Left side of prophage region 5, 10: Right side of prophage region 5, 11: Left side of prophage region 6,12: Right side of prophage region 6.

Through predictive analysis of prophages, six prophage regions were identified in the GD82 genome. Among these, prophage region 1 and region 2 were classified as incomplete, prophage region 5 was predicted as questionable, while prophage regions 3, 4, and 6 were determined to be intact. Their basic characteristics are summarized in Table 3, and their genomic locations are illustrated in Figure 6. Targeted PCR amplification was performed using specific primers designed at both ends of the predicted prophage regions. Electrophoresis results of the GD82 sample demonstrated that all six prophage regions yielded amplification products of expected sizes. (Figure 7).

**Figure 8:**
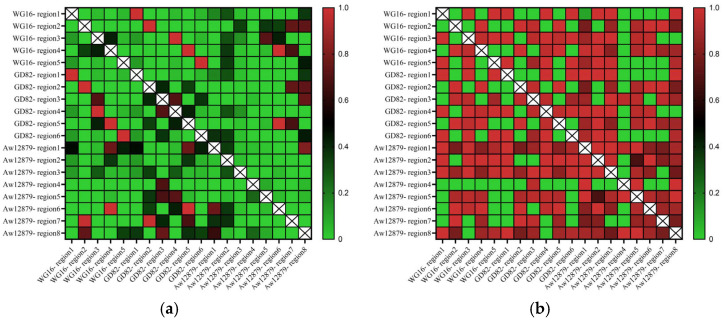
Comparison of prophage region similarity and coverage among different strains. Note: (**a**) Comparison of coverage between strains; (**b**) Comparison of similarity between strains.

The comparison results of the six prophage regions in the GD82 strain genome with the five prophage regions in the WG16 strain and the eight prophage regions in the Aw12879 strain showed that regions 1, 2, 4, and 6 of the GD82 strain had 100% sequence coverage and similarity with regions 1, 2, 3, and 5 of the prophages in the WG16 strain. Region 5 of the GD82 strain had 99.98% sequence similarity with region 4 of the WG16 strain. There was no overlap between region 3 of the GD82 strain and any region of the WG16 strain (Figure 8).

(2) Analysis of Tandem Repeat Sequences in the GD82 Genome

By screening for short tandem repeats (STRs) in the GD82 genome, a total of 273 regions containing tandem repeat sequences were identified. The repeat units ranged from 1.8 bp to 23.7 bp in length, with prediction scores spanning 50 to 1341.

(3) Analysis of Insertion Sequences (IS) in the GD82 Genome

Using the IS Finder database to query the GD82 genome, 20 high-confidence IS elements were detected (Table 4).

(4) Analysis of Genomic Islands in GD82

**Figure 9:**
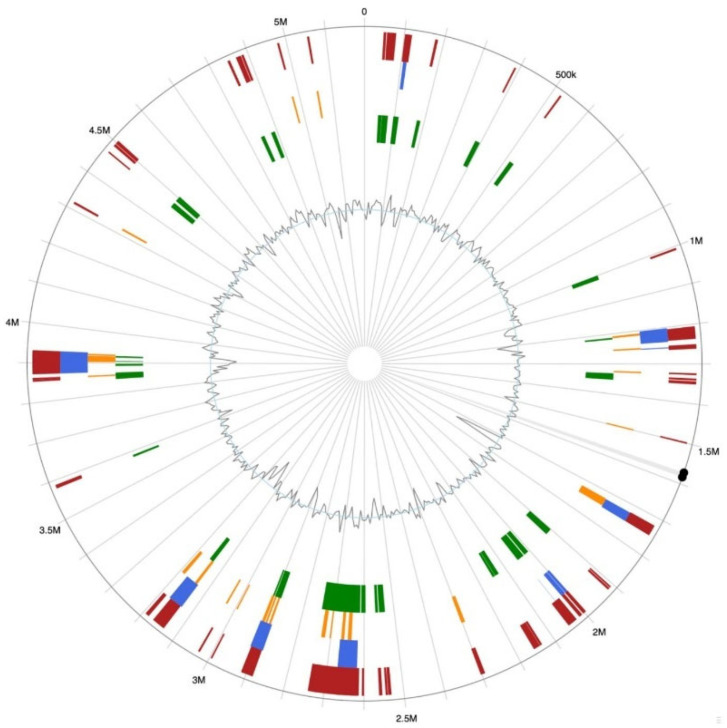
Distribution of gene islands on the genome of strain GD82. Note: The results predicted by IslandPick, SIGI-HMM and IslandPath-Dimob are green, orange and blue, respectively, and the red region is the gene island region obtained by the three methods.

Genomic islands in the GD82 genome were predicted by integrating three methods-IslandPick, SIGI-HMM, and IslandPath-DIMOB-resulting in the identification of 58 genomic islands (Figure 9).

## 3. Discussion

Extracting high-quality DNA from formalin-fixed, historical plant specimens remains a significant challenge, as conventional water-based washing often exacerbates tissue damage [20]. To address this, we developed a novel processing strategy using a neutral PBS buffer. This approach minimizes cellular hydration and structural disruption, thereby substantially improving DNA yield and quality from delicate archival samples. Applying this optimized method, we achieved, to our knowledge, the first genome sequencing of *Xcc* from a Chinese historical herbarium specimen. Although the DNA was highly degraded, our PBS-based protocol enabled the successful analysis of structural variants, plasmid content, and SNP profiles, demonstrating its efficacy for genomic studies of degraded specimens.

Given the highly fragmented and degraded nature of DNA from historical samples, our genomic analysis strategy aimed to extract the maximum amount of information from the available short-read data. We specifically focused on: (i) identifying large structural variations (insertions/deletions) through reference-based mapping; (ii) characterizing plasmid composition by alignment with known plasmid databases; and (iii) conducting comparative single-nucleotide polymorphism analysis to elucidate phylogenetic relationships. Therefore, all comparative conclusions regarding genomic differences and evolution are derived from the aforementioned analyses rather than from complete genome reconstruction. Comparison with the genome of the *Xcc*306 revealed that strain GD82 contains three chromosomal insertion fragments, which were not found in the Guangxi strain U17, Guangdong strain gd2, or Florida strain MN11 [18]. Functional annotation results indicated that these insertion sequences primarily carry genes encoding hypothetical proteins (XAC_RS01495), phage-related genes (*XAC_RS19845*), a helix-turn-helix (HTH) structural protein (XAC_RS19930), andan IS3-like transposase of the ISXac2 family. The abundance of phage-associated sequences in GD82 is notably higher than in many contemporary Xcc genomes, possibly reflecting ancestral genomic content that has been selectively lost in later lineages during adaptation—a pattern consistent with the role of bacteriophages in driving bacterial evolution through horizontal gene transfer and genomic reorganization [21,22,23].

When viewed in a broader geographical and evolutionary context, the structural variations observed in GD82 align with—and help to clarify—recognized region-specific evolutionary trajectories in *Xcc*. For instance, while prophage-related sequences are widely distributed across *Xcc* genomes, their composition and retention appear to vary geographically. The prophage-associated integrase and AlpA-like regulatory genes identified in GD82 show similarities to elements reported in certain Asian and American strains [12], yet the specific combination and genomic location of these insertions in GD82 are distinct. This suggests that while temperate phages may have recurrently contributed to the genome plasticity of *Xcc*, their integration and subsequent evolution have likely been shaped by local ecological and host adaptations.

Compared with published *Xcc* genomes, strain GD82 carries multiple phage-related insertion genes, possibly because it was collected at an earlier time point and retains a higher abundance of phage-associated sequences that were gradually lost during genomic evolution. Previous studies have identified genes related to bacterial adhesion in *Xcc* [24], a critical early step in the pathogenic process. The helix-turn-helix (HTH) domain is present in all prokaryotic genomes and has diverse functions, including DNA repair and replication, RNA metabolism, and protein-protein interactions in different signaling contexts [25]. Comparing the crystal structures of HTH domains across different genomes can help analyze their functional roles and uncover genomic variations. A key feature of the IS3 transposon is the presence of an open reading frame (ORF) encoding a transposase [26,27]. These ORFs typically initiate at the start of the “unique” central region of IS3 (ORFA) and extend to near its end (ORFB). The ORF AB fusion product is considered the functional transposase [28]. The deleted fragment in the chromosome of strain GD82 was annotated as an IS3-like element ISXac2 family transposase. Further studies should analyze the roles of ORFA and ORFB in IS3—whether they suppress transposition by binding to the IS3 promoter (thereby blocking transcription) or by interacting with the inverted repeats (IRs, thus preventing transposase-target binding).

Although this study primarily focused on structural variation and genome content characterization of strain GD82, we acknowledge that further comparative analyses could yield additional insights into the evolutionary origins and functional relevance of the observed insertions. Our observation of a unique structural variant (e.g., insertions and deletions), plasmid content, and comparative SNP profile event in GD82 is distinct from the plasmid profiles described by Campos et al. [12], suggesting region-specific evolutionary trajectories in *Xcc*. In particular, the prophage-associated sequences identified in GD82, such as those encoding integrase and AlpA-like regulatory proteins, may share similarity with elements found in other *Xcc* genomes or citrus-associated microbial taxa. While a comprehensive BLAST-based homology search was not within the scope of the current work, we recognize the value of such analyses and intend to explore these elements in more detail in future studies.

Additionally, we observed an insertion region that harbors a truncated gene with partial homology to TAL effector sequences. Due to the absence of a complete repeat array or canonical activation domains, the functional capacity of this element remains uncertain. Nevertheless, its presence raises intriguing possibilities regarding past acquisition events or gene decay processes, and future work will aim to reconstruct its evolutionary context and assess any potential residual activity.

In this study, we identified several genomic structural variations and prophage insertions unique to the historical strain GD82 of *Xcc*. While these findings offer intriguing insights, interpretations regarding the putative function of AlpA and the ancestral retention of prophage regions remain speculative due to the lack of direct experimental validation.

Furthermore, to better situate our results within a broader evolutionary context, we compared the identified genomic features with those reported in globally diverse *Xcc* strains. Previous studies have documented considerable genomic plasticity among strains from different geographic regions, often linked to variations in virulence factors and host adaptation [12,13]. The prophage elements and insertion regions observed in GD82 may reflect lineage-specific evolutionary trajectories, possibly contributing to pathogen diversity and adaptation over time. Future comparative genomic and functional studies involving a wider range of historical and contemporary strains will be essential to elucidate the evolutionary dynamics and biological significance of these genomic variations.

Future studies integrating more historical genomes from China and adjacent regions will be essential to test these observations, to determine whether the genomic signatures seen in GD82 are representative of earlier Chinese *Xcc* populations, and to further elucidate the functional implications of these lineage-specific variations in pathogenicity and environmental fitness.

## 4. Materials and Methods

### 4.1. Strain Sources

The sweet orange leaves (designated as strain GD82) with typical canker disease symptoms were collected in Guangzhou City, Guangdong Province, China, in 1982 and are currently preserved in the College of Plant Protection, South China Agricultural University. The WG16 strain was isolated from canker-infected leaves of ‘Orah’ (a mandarin hybrid, *Citrus reticulata* cv. Orah) collected in Wuming, Nanning, Guangxi Province.

### 4.2. Pretreatment of Historical Specimen Samples

The pretreatment of citrus canker specimens was modified based on the method described by Xu et al. [29]. Historical DNA extraction from citrus samples was performed using the E.Z.N.A.™ HP Plant DNA Kit (200) (2485-02, OMEGA, Atlanta, GA, USA). The quality of DNA samples from strain GD82 was evaluated by 1% agarose gel electrophoresis and the NanoDrop One spectrophotometer (Thermo Fisher Scientific, Waltham, MA, USA).

### 4.3. Pcr Detection of Gd82 Strain

The primers JYF5/JYR5 (5′-TTCGGCGTCAACAAAATG-3′, 5′-AACTCCAGCACATACGGGTC-3′) reported by Wang et al. [30] were used for conventional PCR detection of strain GD82. The total PCR reaction volume was 20 μL, containing 10× Buffer (2 μL), dNTPs (2 μL, 2.5 mmol/L), forward and reverse primers (0.5 μL each, 10 μmol/L), Taq DNA polymerase (0.2 μL, 2.5 U/μL), DNA template (1 μL, 10 ng), and ddH_2_O (13.8 μL). The PCR cycling conditions were: initial denaturation at 94 °C for 3 min; 32 cycles of 94 °C for 30 s, 54 °C for 30 s, and 72 °C for 45 s; and a final extension at 72 °C for 7 min. PCR products were electrophoresed on a 1% agarose gel (0.5× TBE buffer), stained with Goldview Nucleic Acid Gel Stain (10,000×) (10201ES03, Yeasen, Shanghai, China), and visualized under a gel imaging system. Images were captured and stored for analysis.

The primers XACF07/XACR07 (5′-GAGTCGCCTACCGAGAAATCC-3′, 5′-GACCACGGCAGGGTGAAGA-3′) reported by Yin et al. [31] were used for real-time PCR. The reaction mixture (20 μL) consisted of ddH_2_O (10 μL), forward and reverse primers (0.5 μL each), and blastaq™ 2 × qPCR Master Mix (8 μL). The cycling conditions were: initial denaturation at 95 °C for 5 min; 40 cycles of 95 °C for 10 s and 60 °C for 30 s. Each sample was analyzed in triplicate, and the final Ct values were calculated as the average of three replicates. All primers were synthesized by commericial company.

### 4.4. Illumina Sequencing and Assembly of Gd82 Strain

**Figure 10:**
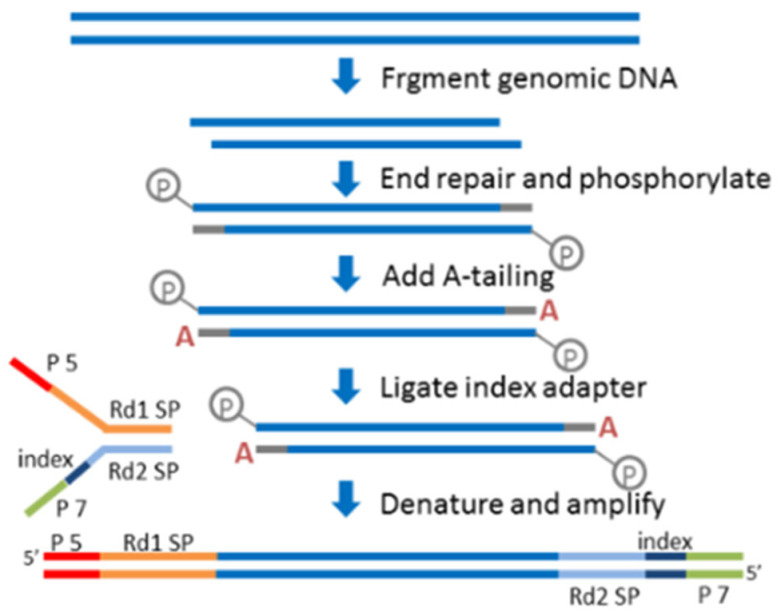
Schematic diagram of library construction. Note, ‘A’ represent Adenine. ‘P’ represent phosphate group. ‘P5’and ‘P7’ are the names of two types of sequencing adapters. ‘Rd1 SP ‘and ‘Rd2 SP’ are primer binding sites in Illumina sequencing adapters. ‘index ‘is a short, specific nucleic acid sequence. ‘blue lines’ represent DNA strands.

The Illumina sequencing library was prepared using the NEBNext^®^ Ultra™ II DNA Library Prep Kit (E7645S, Ipswich, Massachusetts, USA, following the manufacturer’s instructions. This method uses a blunt-end repair and A-tailing strategy followed by T-A adapter ligation, and includes a double-sided size selection step to enrich for fragments between 250–450 bp prior to PCR enrichment. A total of 35 PCR cycles were used during library amplification (Figure 10).

The raw data processing workflow was clarified as follows: reads underwent quality assessment using FastQC v0.11.9, followed by adapter trimming and low-quality base removal, which were performed using fastp (v0.23.2). Reads shorter than 30 bp after trimming were discarded. Importantly, PCR duplicates were identified and removed using Picard MarkDuplicates (v2.26.10), which we acknowledge is essential to avoid inflated depth and erroneous variant calling in degraded samples.

For samples, the HiSeq data were filtered with the genome sequences of *Citrus sinensis* (AJPS00000000.1), C. sinensis mitochondrion (NC_037463.1), and C. sinensis chloroplast genome (DQ864733.1) by using Bowtie2 software (v2.5.2). All mapped reads were removed, and only the unmapped reads were retained for further assembly.

De novo assembly was performed by CLC Genomic Workbench v9.5 (QIAGEN Bioinformatics, Aarhus, Denmark) with default settings. The ordering of *Xcc* contigs was performed by using all de novo assembly contigs to BLAST v2.15.0 against the *Xcc* strain 306 genome (NC_003919.1) through standalone BLAST software (word_size = 28, e-value = 1 × 10^−5^) [32]. For plasmids region, the reference-guided assembly was performed by using three phage sequences (pXAC33, pXAC64, pXAC47 and pLJ207-7.1) as references. The draft *Xcc* genome sequence was generated from the combination of de novo assembly and reference-guide assembly.

### 4.5. Bioinformatic Analysis of Gd82 Strain

**Figure 11:**
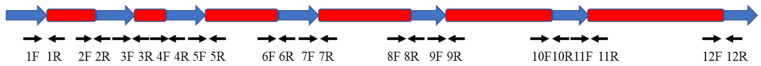
Design of prophage primer on the genome of GD82 strain. Note: The blue arrows represent phage sequences, and the red boxes represent genomic sequences.

Genome annotation was performed using Glimmer v3.02, GeneMarks v4.28, tRNAscan-SE v2.09, and RNAmmer 1.2 [33,34,35,36]. Prophage sequences were predicted using the PHASTER web server (https://phaster.ca/) [37], with subsequent validation through primer design targeting prophage regions and flanking genomic sequences (Figure 11, Table 5). Tandem repeat sequences (TRS) and insertion sequences (IS) were identified using specialized online tools [38]. Genomic islands (GIs) were predicted through IslandViewer4 software [39], which integrates three complementary methods: IslandPick, SIGI-HMM, and IslandPath-DIMOB [40]. For comparative genomics, whole-genome alignments were conducted against published *Xcc* strains (*Xcc*306, NC_003919.1; WG16, CP062255.1) using Standalone BLAST- 2.7.1 [32] with parameters set to word size = 1000 bp and e-value = 0.1. Genome-wide comparative visualization was generated using CGView v1.0 software [41] with the same word size parameter. The phylogenetic tree was constructed using iq-tree on a Linux system with the default parameters.

## 5. Conclusions

In conclusion, we successfully recovered and assembled the first complete genome of *Xcc* strain GD82 from a 1982 formalin-fixed citrus specimen—the oldest genomic record of this pathogen documented in China. Genomic analysis of the historical GD82 strain revealed significant structural variations (including chromosomal insertions/deletions) and prophage integrations, offering critical insights into the evolutionary trajectory of this key citrus pathogen. These findings deepen our understanding of the genetic diversity and adaptive mechanisms underlying citrus canker pathogenicity. Documenting such genomic changes in historical isolates provides a foundation for future studies on pathogen evolution and virulence factors and may ultimately inform the development of more effective disease management strategies. Importantly, functional characterization of these genomic elements remains essential to fully resolve their biological roles and epidemiological impacts.

## Figures and Tables

**Table 1:** Mapping result of *Xcc*-GD82.

Reference	306	pXAC33	pXAC64	pXAC47	pLJ207-7.1
(size in bp)	5,175,554	33,700	64,920	47,152	213,743
Total mapped reads	38,352,851	917,547	1,513,635	805,169	1,754,451
Reference coverage (%)	99%	100%	100%	97%	90%
Average nucleotide coverage (X)	1111	4080	3493	2600	1231
Consensus (bp)	5,136,479	33,703	64,923	47,928	192,938

**Table 2:** General information on *Xcc*-GD82 from historical specimens.

Type	Feature
Genome (bp)	5,197,184
GC content (%)	64.8
Protein-coding gene	4763
tRNA	54
ncRNAs	40
rRNA	6
Plasmids	4

**Table 3:** Basic features of the genomic prophage region of the *Xcc*-GD82 genome.

Prophage Region	Number of CDS	Location	Predicted Status	Region Length (kb)	GC % Content
1	20	1,208,416–1,228,153	incomplete	19.7	59.15%
2	10	1,742,551–1,755,253	incomplete	12.7	63.28%
3	36	2,020,050–2,047,198	intact	27.1	64.07%
4	21	2,539,447–2,565,056	intact	25.6	60.04%
5	11	2,883,792–2,912,086	questionable	28.2	58.42%
6	35	3,146,597–3,192,881	intact	46.2	60.28%

**Table 4:** Basic information of the IS element in *Xcc*-GD82.

WG16-IS	IS Family	IS Length (bp)	E. Value
TnXax1	Tn3	8945	0
ISXc4	Tn3	6938	0
ISXac1	IS4	1350	0
ISXaca1	IS4	1350	0
ISXac3	IS3	1234	0
ISXac4	IS3	1202	0
ISXac2	IS3	1195	0
ISXo14	IS4	1352	0
ISXo4	IS5	1513	0
ISXo9	IS5	1513	0
ISXca5	IS5	1514	0
ISXcd1	IS3	1195	0
ISXoo4	IS5	1530	0
IS1114	IS5	1519	0
ISPsy42	Tn3	5667	0
ISPsy30	Tn3	5167	0
IS1478	IS5	1207	0
IS1481A	IS4	1350	0
ISXc6	IS5	1500	0
ISXca2	IS3	1200	0

**Table 5:** Primer information at both ends of the prophage and genome junction.

Primer Name	Sequence (5′-3′)	Annealing Temperature	Length (bp)
GD82-1F	GGTACGGTAGGAAGCAGGTT	55	521
GD82-1R	ACCATCGGTCAGTGTCAAGT		
GD82-2F	AGGGAGGTTGATTCGGACTC	53	568
GD82-2R	CCTGAAACTGGTCGCACATT		
GD82-3F	CATTTCTCCCATTTCCCGGC	55	614
GD82-3R	GATTTGCCAGCCTATCAGCC		
GD82-4F	CCGAAGCACCTCAACATCAA	55	656
GD82-4R	TCTTCATCCGACAAACCCGA		
GD82-5F	CTGCACTGATTGAGGTCACG	53	749
GD82-5R	CACCCAGACCAAGGACATCA		
GD82-6F	GGCAGCAGATTGTCCAGATG	53	744
GD82-6R	CGTTTAGAACACTGGCGGAG		
GD82-7F	ATTTCAAGGAGTTTCGCCCG	55	403
GD82-7R	CGGTGTTTGATACGGAGCTG		
GD82-8F	GCTGATGCCATTCCCTGATT	53	453
GD82-8R	AGCGAGCGATTCATTGGTTT		
GD82-9F	GCAAGGAAGTGGTCAAGAGC	52	651
GD82-9R	TCGTTGACTCTGCCTCTGTT		
GD82-10F	ATGCGATTTTGATGGGCTGG	55	666
GD82-10R	TTCTTGCTCATGGTGTGGGT		
GD82-11F	GATTTCTCCACCAAGCGACG	55	750
GD82-11R	GGTGCCTTACATCCCCTTCT		
GD82-12F	GCCCAGAAACTCCAAGGTCT	55	781
GD82-12R	GGGATTACGTTGGCTGTTCC		

## Data Availability

The original sequencing data have been submitted to the NCBI database and received GenBank accession number SRR34644352. The data used in this study are already entirely in the public domain (https://www.ncbi.nlm.nih.gov).

## Supplementary Materials

The following supporting information can be downloaded at https://www.mdpi.com/article/10.3390/ijms27020702/s1.
